# Therapeutic safety and efficacy of triple-immunosuppressants versus dual-immunosuppressants in severe-to-critical COVID-19: a prospective cohort study in Bangladesh

**DOI:** 10.1080/07853890.2022.2039958

**Published:** 2022-03-03

**Authors:** Md Jahidul Hasan, Raihan Rabbani, Ahmad Mursel Anam, Shihan Mahmud Redwanul Huq

**Affiliations:** a Clinical Pharmacist (Critical Care and Infectious Diseases/Stewardship), Coordinator-Clinical Pharmacy, Department of Pharmacy, Square Hospitals Ltd, West Panthapath, Bangladesh; b Internal Medicine and Intensive Care Unit, Department of Medical Services, Square Hospitals Ltd, West Panthapath, Bangladesh; c High Dependency Unit (HDU), Department of Medical Services, Square Hospitals Ltd, West Panthapath, Bangladesh

**Keywords:** COVID-19, cytokine storm, immunosuppressant, baricitinib, tocilizumab, secukinumab

## Abstract

**Background:**

Hyperinflammation-induced respiratory failure is a leading cause of mortality in COVID-19 infection. Immunosuppressants such as, Baricitinib and interleukin inhibitors are the drug-of-choice to suppress cytokine storm in COVID-19. Here, we compared the therapeutic safety and efficacy of triple-immunosuppressants with dual-immunosuppressants in patients with severe-to-critical COVID-19.

**Methods:**

This study was conducted on 103 confirmed COVID-19 patients. Of 103 patients, 49 (N) and 54 (N) patients received dual-immunosuppressants (baricitinib plus two doses of secukinumab) and triple immunosuppressants (baricitinib plus single dose of tocilizumab and secukinumab) in group A and group B, respectively. Groups were compared in terms of clinical outcome, critical support-requirement, survival, re-hospitalisation, and adverse events (AEs).

**Results:**

Patients in group B achieved normal blood oxygen saturation level (SpO_2_) earlier than the patients of group A [4 day (IQR: 3–12) vs 5 day (IQR: 5–14), *p* < .05]. The requirement of intensive care unit (ICU) and mechanical ventilation (MV) support was less in group B than group A [16.7%/28.6%, 11.1%/18.4%, respectively *p* < .05]]. The incidence of COVID-19 acute respiratory distress syndrome and 60-day all cause mortality was reduced in group B compared to group A [0.43 (0.19–0.98), *p* < .05; 0.35 (0.08–1.44), *p* > .05]. The 60-day re-hospitalisation rate was two-fold high in group A than group B (*p* = .024). Immunosuppressant-associated adverse events and secondary bacterial/fungal infections were relative high in patients of group B.

**Conclusions:**

Triple-immunosuppressants in severe-to-critical COVID-19 infection exhibited better clinical outcome; reduced ICU and MV requirement; shorter hospital stay with deceased 60-day all cause mortality and re-hospitalisation compared to dual-immunosuppressants.

## Introduction

Severe Acute Respiratory Syndrome Coronavirus-2 (SARS-CoV-2), a novel single-stranded RNA enveloped virus, first spotlighted in Wuhan, China in 2019 through cluster of outbreaks and on 11th March, 2020, World Health Organisation (WHO) declared the associated novel coronavirus disease 2019 (COVID-19) as a pandemic thereat for global human health. Based on recent published data, SARS-CoV-2 is transmitted *via* respiratory droplets, aerosols, and direct contact of infected surface [1,2]. As of 19 October 2021, 240,631,670 confirmed COVID-19 cases and associated 4,899,169 deaths have been reported in 220 countries [3]. Infection and death associated with new strains of SARS-CoV-2 is a rising headache in South Asia including, Bangladesh, and up to 11 October 2021, 15,62,359 confirmed cases and 27,688 deaths have been occurred in Bangladesh [4].

Mortality in hospitalised patients with severe COVID-19 is high worldwide. Recent data shows that mortality rate in hospitalised patients with severe COVID-19 pneumonia symptoms is 8–21%, and up to 78% of patients require support of intensive care unit (ICU) [5–8]. Patients having the sign of respiratory failure with/without multiple organ dysfunction syndromes in addition to severe COVID-19 symptoms are considered as developing severe-to-critical COVID-19 infection [9]. Epidemiological studies showed that 6–10% of patients with severe COVID-19 develop the critical form of the disease, require supports in ICU [10], and mortality rate ranges from 50 to 65% in this group of patients [8,11,12] but, eventually, mortality rate may increase up to 97% in patients with severe-to-critical COVID-19 requiring mechanical ventilation (MV) support [13].

Immune dysregulation and hyperinflammation in severe COVID-19 is a leading cause of high rate of morbidity and mortality worldwide [14]. High expression of pro-inflammatory mediators, including interleukin (IL)-2, IL-6, IL-7, IL-17A, and tumour-necrosis factor-α (TNF-α) in patients with severe-to-critical COVID-19 pneumonia is the major cause of triggering cytokine storm which may result in acute respiratory distress syndrome (ARDS) leading to respiratory failure [8,11,14,15]. On 21 April 2020, the Food and Drug Administration (FDA) of the United States has approved IL-6 inhibitors with dexamethasone in COVID-19 [9]. However, studies on off-label use of monoclonal antibodies (MCAs), including tocilizumab (IL-6 inhibitor), secukinumab (IL-17A inhibitor), sotrovimab (binds with spike protein of SARS-CoV-2), bamlanivimab (binds with spike protein of SARS-CoV-2), baricitinib (janus kinase (JAK) inhibitor that block JAK-1 and JAK-2), and so on in COVID-19 have demonstrated promising outcome in mortality reduction and prevention of MV but therapeutic safety and efficacy data are limited [15–17]. An acute systemic hyperinflammatory response in severe SARS-CoV-2 infection causes acute respiratory distress syndrome (ARDS), and patients with COVID-19 ARDS symptoms urgently require ICU support and specific medication therapies, including steroids and potential immunosuppressants [18]. Data on the use of two or three immunosuppressants jointly along with steroids in severe-to-critical COVID-19 is limited [15,17]. In severe-to-critical stage of COVID-19 infection, while respiratory function is highly impaired due to rapid hyperinflammatory response and conventional therapeutic armamentarium are seems to be vain, and life is in danger, multiple studies mentioned that united therapeutic approach with two or more immunosuppressive agents may be beneficial in obtaining better immunosuppression, improve survival, and reduce ICU or MV support requirement [15–17].

This study aims to evaluate the therapeutic safety and efficacy of double versus triple-immunosuppressants (JAK-1/2 inhibitor plus IL-17A inhibitor versus JAK-1/2 inhibitor plus IL-6 inhibitor plus IL-17A inhibitor) in terms of clinical outcome, survival, ICU and MV requirements, and re-hospitalisation in patients with severe-to-critical COVID-19 pneumonia.

## Materials and methods

### Study design and data collection

This prospective cohort study was conducted from 1 April 2021 to 31 May 2021 on 103 adult patients (≥18 years) with severe-to-critical COVID-19 pneumonia who admitted to the “Specialized COVID-19 Unit” of Square Hospital Ltd, Dhaka, Bangladesh (a tertiary care 400-beded private hospital). With confirmed severe COVID-19 infection, 127 patients were admitted in the hospital during the above mentioned study period. Following the sample inclusion and exclusion criteria of the study, 114 patients were primarily included in the study. Among them, 11 patients were excluded during the study period due to either discharged from the hospital against medical advice or disagreed to take immunosuppressant therapies including tocilizumab and secukinumab. Finally, 103 patients completed the study. No specific sample size was fixed for the study. Admission of the patients with positive COVID-19 test was accomplished through a two-step triage system in the emergency department of the hospital. COVID-19 in patients was confirmed by positive reverse transcriptase polymerase chain reaction (RT-PCR) assay (instrument/device: Rotor Gene-Q/Cobas z480, and QIAGEN kits for real-time PCR, QIAGEN GmbH, Germany) of two separate specimens (nasal and oral swabs) in the Molecular laboratory of the hospital. Clinical diagnosis, comorbidities, lab and microbiological investigations in all patients were evaluated and recorded at the time of hospital admission.

All the patients’ data and history of medications were collected both from the electronic patient database of the hospital and manually from the patients’ medical record file. Lab investigations, assessment of physical status, and drug-associated adverse events in all patients were monitored and recorded daily by a multidisciplinary expert team. The research related to human use has been complied with all the relevant national regulations, institutional policies, and in accordance with the tenets of the Declaration of Helsinki, and has been approved by the Research Ethics Committee, Square Hospitals Ltd, Dhaka, Bangladesh (no. 2103SH-OR036) on 14 March 2021. Written consent was taken from all participants to enrol in the study.

### Study groups and treatment

Among the total 103 patients, 49 and 54 patients (N) were taken into group A and group B, respectively following the simple random sampling method (using a random number generator software). Patients in group A and group B received baricitinib plus secukinumab (two doses 48 h apart) and baricitinib plus tocilizumab (single dose) plus secukinumab (single dose), respectively. In both the groups, unit dosages of the immunosuppressants were same. Baricitinib (supplied brand: “Baritor 2” film-coated tablet manufactured by Square Pharmaceuticals Ltd., Bangladesh) was given as 4 mg once daily, orally for fourteen days; secukinumab (supplied brand: “Cosentyx” 150 mg pre-filled syringe for subcutaneous injection, manufactured by Novartis Pharmaceuticals Corporation, United States) was given as 300 mg, intravenously; and tocilizumab (supplied brand: “Actemra” 200 mg injection vial, manufactured by F. Hoffmann-La Roche AG, Switzerland) was given as 8 mg/Kg of body weight (≤800 mg per dose), intravenously. In both groups, baricitinib therapy was common and started within 6 h of hospital admission. Tocilizumab and secukinumab dosing time (for group B) was separated by 48 h. Each dose of secukinumab and tocilizumab was mixed with 100 mL of 0.9% Sodium chloride solution and administered over 1 h. All the patients also received steroid (dexamethasone: 0.25 mg/Kg of body weight in two divided doses, intravenously; not exceeding 20 mg/day), remdesivir (200 mg as loading dose followed by 100 mg once daily, intravenously for 10 days), low molecular weight heparins (enoxaparin/dalteparin) for anticoagulation, and antibiotics (moxifloxacin or azithromycin) or antifungals (itraconazole or fluconazole) for infections. In case of patients getting discharged to home before completing the 14-day course of baricitinib therapy, the remaining dosages were given to patients at home. No national guidelines in Bangladesh recommended the use of these immunosuppressants except baricitinib and dexamethasone in COVID-19 at the time of the study. The COVID-19 therapeutic review committee jointly with the Research Ethics Committee of the hospital recommended the use of dual or triple immunosuppressants concomitantly with dexamethasone in patients with COVID-19 infection admitted to the hospital under the study protocol.

### Inclusion criteria

Sample inclusion criteria were as follows:

- SARS-CoV 2 is present in the nasal/oral swabs
- No previous history of COVID-19 infection
- Having at least two signs of severe COVID-19 pneumonia with confirmed pneumonia lesions (bilateral ground-glass opacities) (>50%) in the chest computerised tomography (CT) scan images: (I) dyspnoea; (II) oxygen saturation in peripheral blood (SpO_2_) level ≤93% on room air at sea level; and (III) respiratory rate ≥30 breaths/min
- Having at least one sign of critical COVID-19 pneumonia in addition to severe COVID-19 symptoms: (I) SpO_2_ <90% on maximum support of high flow nasal cannula (HFNC); (II) sign of sepsis or septic shock; (III) one or more organ dysfunction
- Duration of onset of symptom(s)-to-hospitalisation is no more than 10 days

### Exclusion criteria

Sample exclusion criteria were as follows:

- History of taking vaccination for COVID-19
- Patient with current pregnancy, history of malignancy, active tuberculosis, organ transplantation, and smoking
- Any history of trauma or elective surgical procedure within the last 3 months of onset of COVID-19 symptoms
- Any history of acute/chronic autoimmune disease, active/latent tuberculosis infection, and taking any immunosuppressant drug
- Current evidence of bacterial or fungal coinfection
- History of taking any anti-inflammatory drug within last 3 months of hospital admission

### Definitions and statistical analysis

Clinical characteristics of severe COVID-19 pneumonia include oxygen saturation in peripheral blood capillary (SpO_2_) <94% on room air at sea level, a ratio of arterial partial pressure of oxygen to fraction of inspired oxygen (PaO_2_/FiO_2_) <300 mm Hg, respiration rate >30 breaths/min, or lung infiltrates >50% in CT scan of chest. Critical COVID-19 is defined as having at least one of the following conditions in addition to existed severe COVID-19 symptoms: (a) respiratory failure on maximum supplemental oxygen support by HFNC and requiring mechanical ventilation in an ICU setup; (b) sign of sepsis or septic shock; (c) developing one or multiple organ failure [9]. COVID-19 ARDS can be defined as confirmed COVID-19 infection with the Berlin 2012 ARDS criteria, including (i) acute hypoxaemic respiratory failure; (ii) developed within 1 week of respiratory dysfunction; (iii) bilateral pulmonary airspace on chest x‐ray or CT scan or chest ultrasound; and (iv) no cardiac involvement in aggravating acute hypoxaemic respiratory failure [18]. Secondary infection in patients with COVID-19 can be defined as an infection caused by bacteria or fungus developed during or after COVID-19 infection in the hospital [19].

The statistical analyses were performed by Statistical Product and Service Software (SPSS ver. 22.0, Chicago, IL, USA). Multivariable logistic regression analysis was done to calculate odds ratios (ORs) with 95% confidence intervals (CI). Descriptive statistics were presented through median value and interquartile range (IQR). Continuous variables were compared using Mann–Whitney U test, and categorical variables were compared using Pearson Chi-square test. To analyse 60-day survival among the groups (dual-immunosuppressants versus triple-immunosuppressants), we plotted Kaplan-Meier curves. A *p* value of ≤.05 was considered statistically significant.

## Results

In group A, 49 patients were treated with dual-immunosuppressants (4 mg, once daily oral dose of baricitinib for 14 days/2 intravenous dosages of secukinumab) and 54 patients in group B received triple-immunosuppressants (4 mg, once daily oral dose of baricitinib for 14 days/one intravenous dose of tocilizumab/one intravenous dose of secukinumab). The number of male patients in both the groups was higher than the number of female patients with a median age of 53 (IQR: 46–56.5) and 55 (IQR: 46–56.5) in group A (*N* = 49) and group B (*N* = 54), respectively (*p* = .021). The median time from onset of symptoms to hospitalisation and treatment initiation was within 7 days and 9 days in both the groups, respectively. With fever [101 (IQR: 99–101.5)/101 (IQR: 99.5–102) (*p* = .212)], other symptoms, including dry cough (89.79%/94.44%) (*p* = .773), weakness (93.87%/94.44%) (*p* = .708), dyspnoea (91.84%/96.29%) (*p* = .823), headache (79.59%/88.89%) (*p* = .802), anosmia (87.76%/90.74%) (*p* = .800), diarrhoea (44.89%/57.41%) (*p* = .844), and sore throat (55.1%/38.88%) (*p* = .744) were diagnosed in patients of group A (*N* = 122) and group B (*N* = 116), respectively. Diabetes, hypertension, ischaemic heart diseases (IHD), bronchial asthma (BA), chronic kidney disease (CKD), chronic obstructive pulmonary disease (COPD), obesity, peptic ulcer disease (PUD), chronic liver diseases (CLD), and Parkinson’s disease were found as comorbidities in all patients of the study (group A and B) and mentioned in detail in Table 1. The median SpO_2_ was 90% in both the groups, and the ratio of arterial oxygen partial pressure to fractional inspired oxygen (PaO_2_/FiO_2_) was 257 mm Hg (IQR: 214–286.5) and 260 mmHg (IQR: 209.5–289) in group A and group B, respectively (*p* > .05). The requirement of supplemental oxygen was similar in both groups. Other clinical characteristics, including respiratory, cardiac, kidney, and liver functions, infection markers, inflammation marker, and hematological components of all the patients in both the groups are given in Table 1 and compared, statistically. The median Modified Early Warning Score in patients of both groups was 3 (IQR: 3–4) (*p* > .05) and considering that score, patients were taken under close clinical observation.

**Table 1.** Baseline demographic information, symptoms of COVID-19, comorbidity and clinical characteristics in patients of the study.

Variable	Group A (*N* = 49)	Group B (*N* = 54)	*p* value
Male/female, *n* (%)	29/20 (59/41)	31/23 (57/43)	.477
Age (year), median (IQR)	53 (46–56.5)	55 (47.5–60)	.021
Onset of symptom-to-hospitalisation time, median (IQR)	6 (4–7)	7 (5–7.5)	.402
Onset of symptom-to-drug therapy, median (IQR)	9 (9–11)	9 (9–11.5)	.370
Symptom			
Fever (°F), median (IQR)	101 (99–101.5)	101 (99.5–102)	.212
Dry cough, *n* (%)	44 (89.79)	51 (94.44)	.773
Weakness, *n* (%)	46 (93.87)	51 (94.44)	.708
Dyspnoea, *n* (%)	45 (91.84)	52 (96.29)	.823
Headache, *n* (%)	39 (79.59)	48 (88.89)	.802
Anosmia, *n* (%)	43 (87.76)	49 (90.74)	.800
Diarrhoea, *n* (%)	22 (44.89)	31 (57.41)	.844
Sore throat, *n* (%)	27 (55.1)	21 (38.88)	.744
Comorbiditity			
Diabetes, *n* (%)	40 (81.63)	39 (72.22)	.872
Hypertension, *n* (%)	19 (38.78)	24 (44.44)	.886
IHD, *n* (%)	18 (36.73)	16 (29.63)	.888
Bronchial asthma, *n* (%)	5 (10.20)	4 (7.41)	.667
CKD, *n* (%)	14 (28.57)	15 (27.78)	.751
COPD, *n* (%)	3 (6.12)	2 (3.70)	.798
Obesity, *n* (%)	19 (38.78)	23 (42.59)	.961
PUD, *n* (%)	10 (2.04)	17 (31.48)	.886
CLD, *n* (%)	6 (12.24)	3 (5.56)	.847
PD, *n* (%)	3 (6.12)	4 (7.41)	.883
Clinical characteristics			
SpO_2_ (%), median (IQR)	90 (89.5–90)	90 (88–90)	.885
PaO_2_/FiO_2_ (mmHg), median (IQR)	257 (214–286.5)	260 (209.5–289)	.784
RSO, median (IQR)	6 (4.5–7)	7 (5–8.5)	.921
Respiratory rate, (breaths/min), median (IQR)	24.5 (21–26.5)	25 (22.5–26)	.687
Heart rate (beat/min), median (IQR)	98 (84–106.5)	94.5 (86–19.5)	.430
CRP (mg/L), median (IQR)	168.5 (88.5–219)	146 (54.5–232)	.988
Procalcitonin (ng/mL), median (IQR)	2.12 (1.04–3.98)	1.27 (0.79–1.22)	.400
WBC (K/µL), median (IQR)	8.6 (5.3–14.61)	9.41 (6.2–12.52)	.644
Neutrophils (%), median (IQR)	88.1 (76.8–92.48)	79.8 (83.4–90.58)	.897
Lymphocytes (%), median (IQR)	11.9 (10.19–14.65)	12.9 (9.25–14.85)	.709
Platelet (K/µL), median (IQR)	116 (88.2–179)	146.1 (49–217.3)	.438
D-dimer (mg /L FEU), median (IQR)	3.44 (3.62–7.7)	4.8 (3.99–6.56)	.833
IL-6 (pg/mL), median (IQR)	144 (41.1–188)	154 (62–200.6	.895
Serum Ferritin (ng/mL), median (IQR)	711 (497.5–873)	703.2 (490–736.6)	.840
LDH ((U/L), median (IQR)	629 (514.2–692)	539.4 (411–687.1)	.641
Creatinine (mg/dL), median (IQR)	1.3 (1.19–2.3)	1.2 (1–2.04)	.948
ALT (U/L), median (IQR)	53 (47–72.6)	59 (41.5–79.5)	.692
AST (U/L), median (IQR)	38 (25.5–51.3)	39 (31.5–56.5)	.695
MEWS, median (IQR)	3 (3–4)	3 (3–4)	.994

SNB: secukinumab; BCB: baricitinib; IQR: interquartile range; n: number; %: percentage; F: grade Fahrenheit; IHD: ischaemic heart disease; CKD: chronic kidney disease; COPD: chronic obstructive pulmonary disease; PUD: peptic ulcer disease; CLD: chronic liver disease; PD: Parkinson’s disease; SpO_2_: oxygen saturation in peripheral blood; PaO_2_/FiO_2_: ratio of arterial oxygen partial pressure to fractional inspired oxygen; mmHg: millimetre of mercury; RSO: requirement of supplemental oxygen; min: minute; CRP: C-reactive protein; mg: milligram; L: litre; FEU: fibrinogen equivalent units; ng: nanogram; WBC: white blood cells; K/µL: thousand cells per micro litre; IL: interleukin; pg/mL: picograms per millilitre; LDH: lactate dehydrogenase; U/L: units per litre; dL: decilitre; ALT: alanine aminotransferase; AST: aspartate aminotransferase; MEWS: Modified Early Warning Score.

The median day to achieve the normal SpO_2_ level (≥94% on room air) and get free-from-supplemental oxygen requirement was significantly less in patients of group B than in patients of group A [4 (IQR: 3–12)/5 (IQR: 5–14); 5 (IQR: 4–5)/8 (IQR: 6–9), respectively]. Requirement of ICU and MV support was higher in patients of group A (*N* = 49) compared to group B (*N* = 54) [28.6%/16.7% (*p* = .004); 18.4%/11.1% (*p* = .038), respectively]. The risk of developing COVID-19 ARDS was significantly high in patients of group A compared to group B [OR = 0.43 (0.19–0.98), 95% CI, *p* = .045]. The prevalence of secondary infections in hospital caused by bacteria or fungus was high in patients treated with three different immunosuppressants rather than that in patients treated with two immunosuppressants [OR = 1.2 (0.49–2.99), 95% CI, *p* > 0.05]. The 60–day all-cause mortality rate was higher in patients of group A rather than that in patients of group B [14.29% (*N* = 49) versus 7.41% (*N* = 54), respectively; OR = 0.35 (0.08–1.44), 95% CI, *p* > .05] (Table 3). The median length-of-hospitalisation (LOH) was 10 days (IQR: 9–12) in group A whereas LOH was 15 days (IQR: 14–18) in group B (Table 2).

**Table 2.** Clinical outcomes in patients with severe COVID-19 pneumonia treated with baricitinib plus secukinumab (Group A) or baricitinib plus secukinumab plus tocilizumab (Group B).

Parameters	Group A (*N* = 49)	Group B (*N* = 54)	*p* value
Days for SpO_2_ ≥94% on room air, median (IQR)	8 (6–9)	5 (4–5)	.001
Days for no supplemental oxygen demand, median (IQR)	8 (6–9)	5 (4–5)	.001
ICU support required, *n* (%)	14 (28.6)	9 (16.7)	.004
MV support required, *n* (%)	9 (18.4)	6 (11.1)	.038
Secondary infections			
Bacterial, *n* (%)	6 (12.2)	10 (18.5)	.079
*Klebsiella pneumoniae*	2	3	
*Acinetobacter baumannii*	1	1	
*Pseudomonas aeruginosa*	–	1	
MRSA	–	2	
MSSA	1	1	
*Stenotrophomonas maltophilia*	2	2	
Fungal, n (%)	3 (6.1)	8 (14.8)	.004
*Candida albicans*	2	5	
*Non-albicans Candida* species	1	3	
Length-of-hospitalisation (day), median (IQR)	15 (14–18)	10 (9–12)	.012

HD: high dose; UD: usual dose; SpO_2_: peripheral capillary oxygen saturation; IQR: interquartile range; ICU: intensive care unit; MV: mechanical ventilation; *n*: number; %: percentage; MRSA: methicillin-resistant *Staphylococcus aureus*; *MSSA*: methicillin-susceptible *Staphylococcus aureus*.

**Table 3.** Analysis of risk of therapy in patients treated with dual- versus triple-immunosuppressants.

Parameter	Odds ratio (95% confidence interval)	*p*-value
Development of COVID-19 ARDS^a^	0.43 (0.19–0.98)	.045
Secondary infections (bacterial/fungal)	2.22 (0.88–5.56)	.088
60-day all-cause mortality	0.35 (0.08–1.44)	.148

^a^ Coronavirus disease 2019 acute respiratory distress syndrome.

**Figure 1.**
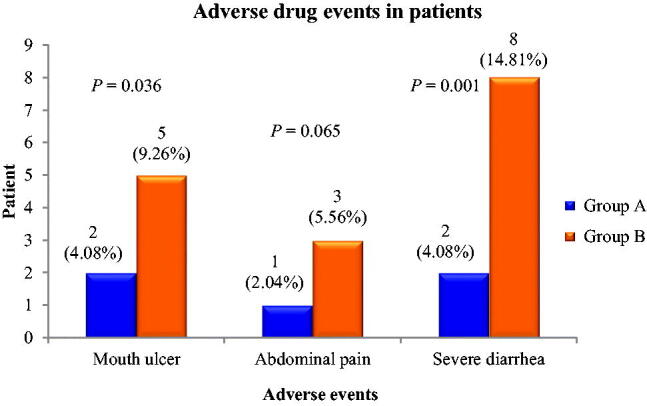
Adverse drug events in patients of group A (treated with baricitinib plus two doses of secukinumab) and group B (treated with baricitinib plus single dose of tocilizumab and secukinumab).

**Figure 2.**
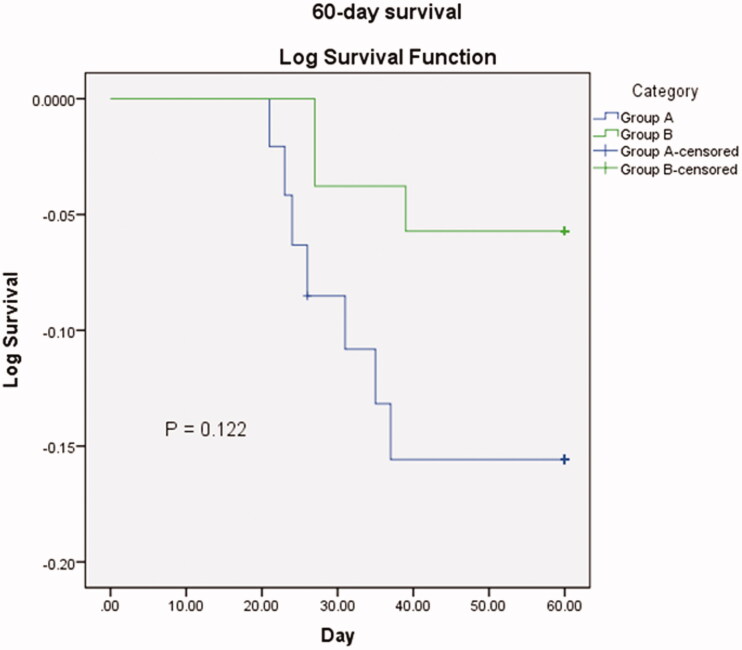
Kaplan-Meier 60-day survival curve for group A (treated with baricitinib plus 2 doses of secukinumab) (blue line) and group B (treated with baricitinib plus one dose of tocilizumab plus one dose of secukinumab) (green line). Analysis was ran using Group (group B/case vs group A/control) as factor; death as event and time to death as time variable.

**Figure 3.**
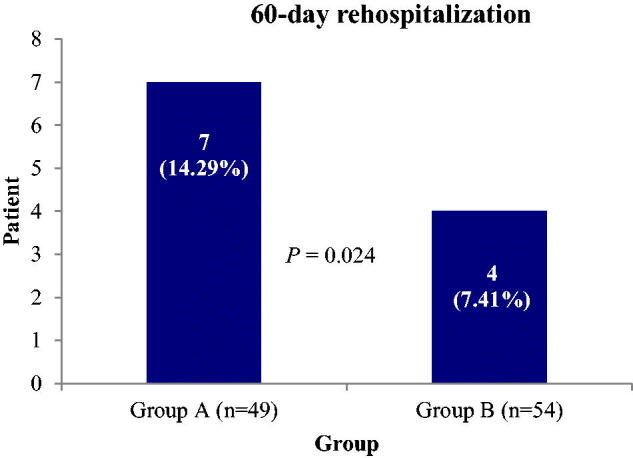
The 60-day rehospitalization of the patients treated with baricitinib plus secukinumab (two dosages) (group A) or baricitinib plus secukinumab plus tocilizumab (group B).

Compared to patients of group A, the incidence rate of adverse events associated with immunosuppressive agents (baricitinib/tocilizumab/secukinumab) in patients of group B was higher, and these included mouth ulcer (4.08%/9.26%, *p* = .036), abdominal pain (2.04%/5.56%, *p* = .065), and severe diarrhoea (frequency was 10–12 times a day) (4.08%/14.81%, *p* = .001) (Figure 1). The complications were resolved with substantial medical management without discontinuing the corresponding drug therapies. The Kaplan-Meier 60-day survival curve was analysed using the study groups (A vs B). Within 60-day of receiving immunosuppressant therapies, around 3-fold higher mortality rate was found in patents of group A (*N* = 49) than the patients in group B [14.29% (*N* = 49) vs 5.56% (*N* = 54), *p* > .05] (Figure 2). Within 60-day after getting discharge from the hospital with acceptable health condition upon medical advice, approximately 2-fold patients in group A were re-admitted to the hospital with respiratory complications than that in patients of group B (*p* < .05) (Figure 3).

## Discussion

In this study, patients with severe COVID-19 pneumonia in group A treated with baricitinib (JAK-1/2 inhibitor) with one dose of tocilizumab (IL-6 inhibitor) plus one dose of secukinumab (IL-17A inhibitor) (48 h apart) showed faster normalisation of the SpO_2_ (≥94% on room air) without supplemental oxygen support, less requirement of ICU and MV support, improved survival rate, and reduced re-hospitalisation rate compared to patients treated with baricitinib plus two doses of secukinumab in group B. The off-label use of immunomodulatory agents, including baricitinib, secukinumab, and tocilizumab in COVID-19 is very challenging because of limited data on safety and efficacy of the therapy [20]. Several studies found that the use of experimental immunosuppressants within early hours of onset of severe COVID-19 symptoms in hospital may prevent further progression of the COVID-19-associated complications, suppress hyperinflammatory responses, reduce ICU and MV supports, and all-cause mortality [6,14–17]. Thus, therapeutic success of these agents, alone or in combination, with other concomitant therapies, enhances the possibility to minimise the severity of the disease [20]. In our study, a combination of three immunosuppressants with variable target-specific action exhibited superior clinical outcome, less re-hospitalisation with reduced mortality than the combination of two immunosuppressants.

COVID-19 infection at severe stage causes lethal lung injury mostly by triggering cytokine storm leading to ARDS, and about one-third of hospitalised patients with COVID-19 symptoms develop ARDS [21]. Several studies found that the extrapulmonary systemic hyperinflammatory syndrome (ESHS), also known as cytokine storm, in COVID-19 which is developed due to over expression of pro-inflammatory cytokines and other mediators in the host body, known as the third-phase of COVID-19 infection, has the pivotal role in potentiating ARDS [12–14,21]. The development of ARDS also linked to the activation of Ang-II pathways which highly trigger-up the adaptive immunity through the excessive release of infiltrating macrophages and other pro-inflammatory mediators [22] leading to over expression of interleukins, tumour necrosis factor α (TNF-α), interferon-gamma (IFNγ), and chemokines (e.g. CXC L10, and CCL2) which results in massive cytokine storm [21,22]. Cytokine storm and other factors, including high level of inflammatory mediators, endothelial dysfunction, coagulation disorder, infiltrated inflammatory cells in different organs, and secondary bacterial or fungal infections increase the risk of dysfunction of multi-organs, including lung, liver, kidney, heart, nervous, gastrointestinal, and hematological system leading to critical phase of COVID-19 infection, and at that stage, ICU and MV support becomes highly required [12,13,23]. An expanded meta-analysis on different geographical regions of the world showed that ICU-mortality rate related to critical COVID-19 ranged from 30 to 40% in most of the regions [24]. ICU mortality rates in patients with severe COVID-19 range from 50 to 67% [11,12,25], and this rate increased up to 97% while patients receiving MV [13]. Another study in Wuhan found that 41.8% of patients with COVID-19 pneumonia developed ARDS and mortality rate was 52.4% [26]. In the current study, 22.33% and 14.56% of patients (*N* = 103) undergoing critical stage of COVID-19 infection required ICU and MV support, respectively; and patients with severe COVID-19 symptoms received triple-immunosuppressants for the suppression of cytokine storm significantly required less ICU and MV supports than the patients treated with dual-immunosuppressants [16.7%/28.6%; 11.1%/18.4%, respectively].

The use of immunosuppresants in hyperinflammation phase of COVID-19 is still a matter-of-debate worldwide. Out-of-debate, JAK inhibitors, including baricitinib, tofacitinib, and ruxolitinib are widely using in COVID-19 [15]. A study found that JAK inhibitors reduced ARDS [risk ratio (RR) = 0.50 (0.19–1.33)], and significantly decreased ICU [RR = 0.24 (0.06, 1.02)], and MV support [RR = 0.63 (0.47–0.84)] [27]. Another study in Bangladesh showed that baricitinib at high dose (8 mg/day) reduced ICU (91%) and MV requirement (95.9%) with a low 30-day mortality rate (3.3%) [28]. Despite the conflicting clinical outcome of tocilizumab in patients with COVID-19 demonstrated in several studies [14–16,20], including an Italian study where no significant difference was found in ICU admission and 7-day mortality rate between treated and untreated groups [29], some other studies found higher survival rate with less ICU and MV requirement in tocilizumab treatment group [30–32]. Maximum two doses of tocilizumab (24–48 h apart) has been given in some studies illustrated improved clinical outcome [31,32]. Data on IL-17A inhibitor, such as secukinumab, in COVID-19 is limited but a recent study showed that the secukinumab-baricitinib combination therapy compared to baricitinib single therapy in COVID-19 showed lower rate of ICU and MV requirement (23.53%/52.94%, *p* = .02; 11.76%/29.41%, *p* = .011) with decreased 30-day mortality rate (5.88%/17.65%, *p* = .033) [17].

Scarcity of data on the use of two or more immunosuppressants with a time-interval in patients with COVID-19 does not give a clear understanding regarding the benefit of amalgamated immunosuppressant therapy in COVID-19 [15,16,20,27]. However, some studies on associated immunosuppressants, such as baricitinib with tocilizumab [33] and baricitinib with secukinumab [17] found superior survival benefits, reduced requirement of ICU and MV support, and declined rehospitalization [15–17,33]. In our study, in patients with severe COVID-19, 4 mg per day baricitinib therapy with single dose of tocilizumab and secukinumab at 48 h interval exhibited better reduction in ICU and MV requirement than similar baricitinib therapy with 48 h apart two dosages of secukinumab.

The humanised anti-IL-6R monoclonal antibody tocilizumab promptly activates the release of proinflammatory cytokines and chemokines, promotes the differentiation of monocytes into macrophages, and thus triggers other immune cells to activate through its trans-signalling pathway while host body detects SARS-CoV-2 as a threat [14,16,31]. The role of IL-6 in hyperactivation of pulmonary inflammatory cascades is significantly synergized by IL-17A [34]. Moreover, the IL-6 mediated Th17 (T helper 17) differentiation system, which promotes more release of IL-6 in turn, is progressed through the JAK-STAT3 (signal transducer and activator of transcription 3) signalling pathway, and blockage of JAK-STAT3 signalling pathway and extinction of IL-17A activity reduces the potentiality of IL-6 through reducing its release and activation [17,33,34]. A study suggested that a combination of IL-6 inhibitor (tocilizumab), IL-17A inhibitor (secukinumab), and JAK inhibitor (baricitinib) may provide optimum suppression of cytokine storm in COVID-19 infection [34]. Similarly, in this study, triple-immunosuppressants (baricitinib plus single dose of tocilizumab and secukinumab) demonstrated shorter period to normalise SpO_2_, minimised hospitalisation time, reduced mortality, and declined 60-day re-hospitalisation with worsen respiratory symptoms (7.41% vs 14.29%) compared to dual-immunosuppressants (baricitinib plus two doses of secukinumab) in patients with severe-to-critical COVID-19.

In several studies, secondary infections were high in patients received immunosuppressants and one of the leading causes of death in COVID-19. In this study, patients received three different immunosuppressants were significantly more susceptible to secondary infections (bacterial/fungal) in hospital compared to patients treated with dual-immunosuppressants [OR = 1.2 (0.49–2.99), *p* < .05]. Immunosuppressants-associated AEs, including sore throat, abdominal pain, and diarrhoea were more frequent in patients received an additional single dose of IL-6 inhibitor to JAK and IL-17A inhibitor (group B; *N* = 54) than the patients received JAK-1/2 inhibitor with double dose of IL-17A inhibitor (group B; *N* = 49) but, all the AEs were well managed with conventional drug therapies and no sequelae of the AEs found. Some studies are highly required to find out the exact mechanism of two or more immunosuppressants jointly in suppressing cytokine storm in COVID-19 infection, and the complete safety and efficacy profile of these potential immunosuppressants in COVID-19. To our knowledge, the major limitations of this study are the small sample size, no post-therapy monitoring of IL-6 and IL-17A level, and absence of therapeutic safety assessment in different comorbidities.

## Conclusion

Immunosuppressants including interleukin and JAK inhibitors are considered as the last-line agent for saving lives of patients with severe COVID-19 infection while conventional treatments become unable to suppress hyperinflammatory response. However, therapeutic safety and efficacy of these agents are still not clear. This study found less ICU and MV demand, reduced 60-day mortality, and declined 60-day re-hospitalisation in patients with severe-to-critical COVID-19 receiving triple-immunosuppressants (baricitinib plus single dose of tocilizumab and secukinumab) rather than dual-immunosuppressants (baricitinib plus two doses of secukinumab).

## Data Availability

Study data are available upon request to corresponding author.

## Abbreviations

COVID-19: Coronavirus disease 2019
SARS-CoV-2: Severe Acute Respiratory Distress Syndrome Coronavirus-2
WHO: World Health Organisation
FDA: Food and Drug Administration
ICU: Intensive care unit
MV: Mechanical ventilation
AE: Adverse event
JAK: Janus kinase
IL: Interleukin
TNF-α: Tumour-necrosis factor-α
ARDS: Acute respiratory distress syndrome
MCA: Monoclonal antibodies
SpO_2_: Oxygen saturation in peripheral blood
CT: Computerised tomography
HFNC: High flow nasal cannula
PaO_2_/FiO_2_: Ratio of arterial partial pressure of oxygen to fraction of inspired oxygen
OR: Odds ratio
CI: Confidence intervals
IQR: Interquartile range
IHD: Ischaemic heart diseases
BA: Bronchial asthma
CKD: Chronic kidney disease
COPD: Chronic obstructive pulmonary disease
PUD: Peptic ulcer disease
CLD: Chronic liver diseases
ESHS: Extrapulmonary systemic hyperinflammatory syndrome
TNF-α: Tumour necrosis factor α
IFNγ: Interferon-gamma
Th17: T helper 17
JAK-STAT3: Signal transducer and activator of transcription 3
